# MRI visibility of the anterolateral ligament and the deep structures of the iliotibial tract

**DOI:** 10.1186/s40634-020-00244-8

**Published:** 2020-05-06

**Authors:** Michael Liebensteiner, Armin Runer, Christof Kranewitter, Philipp Nachtigal, Johannes Giesinger, Dietmar Dammerer, Benjamin Henninger

**Affiliations:** 1 Deptartment of Orthopedic Surgery, Medical University of Innsbruck, Innsbruck, Austria; 2 Deptartment of Trauma Surgery, Medical University of Innsbruck, Innsbruck, Austria; 3 Department of Radiology, Medical University of Innsbruck, Innsbruck, Austria; 4 Medical University of Innsbruck, Innsbruck, Austria; 5 Innsbruck Institute of Patient-centered Outcome Research (IIPCOR), Innsbruck, Austria

**Keywords:** Anterolateral ligament, Iliotibial tract, Iliotibial band, Anterior cruciate ligament, Knee stability, Magnetic resonance imaging

## Abstract

**Purpose:**

The visualization of potentially injured anatomical structures is crucial. Lately the anterolateral ligament (ALL) and the deep structures of the iliotibial tract (ITT) have been of increased clinical interest because of their role as important lateral stabilizers of the knee. The aim of this study was to assess the visibility of the ALL and the deep structures of the ITT using MRI. Good intra- and inter-observer reproducibility was hypothesized.

**Methods:**

Knee MRI data from patients without ligamentous lesions were retrospectively analyzed by two radiologists at two time points using axial and coronal sequences. The visibility of the different parts of the ALL (femoral, meniscal and tibial part) and of the deep ITT, namely the deep attachments of the ITT to the distal femur and capsulo-osseous layer of the ITT, were determined on a binary (yes/no) basis.

**Results:**

Seventy-one cases (42 men, 29 women) were studied. Inter-observer agreement was high. Cohen’s kappa was 0.97 for the tibial part of the ALL and 0.76 for the femoral part. For the deep attachments of the ITT to the distal femur Cohen’s kappa was 0.94. For each of the investigated parameters absolute agreement between the observers was at least 88%. Regarding intra-observer agreement Cohen’s kappa was 0.62 for the femoral part of the ALL and 0.85 for the tibial part of the ALL. For the deep attachments of the ITT to the distal femur Cohen’s kappa was 0.94. For each investigated parameter absolute agreement between the two time points was at least 83%.

**Conclusions:**

The presence of the anterolateral structures of the knee can be determined with substantial inter- and intra-observer agreement using MRI examination. This is applicable for both the ALL and the deep ITT.

**Level of evidence:**

Diagnostic study – Level III.

## Background

Since 2013 there has been an increased interest in the anterolateral extra-articular soft-tissue structures of the knee and their importance in controlling rotatory knee stability. In particular, the anterolateral ligament (ALL) was popularized as an important ligamentous stabilizer of the anterolateral knee [4]. This triggered several investigations of ALL traceability during cadaver dissection [4, 5, 11, 29, 31, 32, 36] and the biomechanical function of the ALL [19, 26, 29, 33, 35]. Several studies have dealt with MRI visibility of the ALL [9, 12–14, 20, 21, 24, 30, 37]. Nonetheless, little is known about validity and reproducibility in assessing the ALL [7, 13, 14, 37]. Ferretti et al. [7] reported inter- and intra-observer reliabilities of the ALL in different MRI imaging parameters ranging between 0.69–1 and 06–1, respectively. Helito et al. [13] studied the validity of MRI in detecting the ALL using an anatomic evaluation as reference and reported an intra-observer reliability between 0.77 and 0.93.

While the above-mentioned authors promote the ALL as a main anterolateral knee stabilizer, others refuted the relevance of the ALL in providing anterolateral knee stability [18] and put more emphasis on the role of the deep portions of the iliotibial tract (ITT). The deep portion of the ITT was first described in the 1960s by Kaplan et al. and consists of two distinct parts: the deep attachments of the ITT to the distal femur and the capsulo-osseous layer of the ITT [17, 23, 28, 38].

This polarity between the deep structures of the ITT and the ALL is also evident in the field of ‘anterior cruciate ligament reconstruction with concomitant anterolateral extraarticular reconstruction’. While some authors have advocated reconstruction of the ACL in combination with anatomical ALL reconstruction [16, 34], others prefer extra-anatomical lateral tenodesis by inserting the graft more proximal at the femur and therefore tend to mimic the deep structures of the ITT rather than those of the ALL [1, 25]. For a more personalized and tailored anatomical reconstruction special care has to be taken to identify the real extent of extra-articular concomitant injuries in order to repair or reconstruct this specific structure, e.g. ALL or deep ITT. However, previous research investigated only MRI visibility of the ALL, providing visibilities between 51% and 98% [3, 14, 37]. To date no such investigations were performed for the deep structures of the ITT.

The aim of the present study was to assess the visibility and reproducibility of both the ALL and the deep structures of the ITT using magnetic resonance imaging. It was hypothesized that Cohen’s kappa values for intra- and inter-observer reproducibility would be seen to be above 0.70, indicating substantial agreement between the ratings [22].

## Materials and methods

Ethics approval was obtained from the Ethics Committee of the Medical University of Innsbruck. The retrospective analysis was conducted in accordance with the ethical standards of the Declaration of Helsinki.

MRI data from patients aged 18 to 40 obtained between January 2017 and December 2018 were retrospectively reviewed. Only healthy knees without any clinically or radiologically diagnosed intra- or extraarticular ligamentous pathology were included. Further exclusion criteria were lesions to the joint capsule, fractures, bone edemas as well as foreign material and motion artifacts. Moreover, medical records were checked for past knee injuries or operative interventions.

All MRIs were obtained using the identical protocol. Patients were examined in supine position using a dedicated 15-channel knee coil. The following sequences were used for our 1.5 T / 3.0 T Scanner (Avanto/Skyra, Siemens, Erlangen, Germany): coronal T1-weighted images (TE 10/13, TR 696/522, SL 3 mm); coronal PD-weighted images with fat saturation (TE 40/38, TR 4100/3230, SL 3 mm); sagittal PD-weighted images with fat saturation (TE 39/38, TR 3000/3710, SL 3 mm) and axial PD-weighted images with fat saturation (TE 31/37, TR 3010/3100, SL 3.5/3 mm).

Two trained and experienced musculoskeletal radiologists (HB, KC) analyzed the pictures using the imaging viewer Impax EE (Agfa Health Care N.V., Mortsel, Belgium). Before commencement, a specialist in the field of anterolateral knee anatomy lectured and briefed both radiologists in a private cadaver dissection class. All relevant lateral and anterolateral structures of the knee were dissected and studied.

Thereafter, each observer performed all the below-mentioned analyses separately. To allow calculation of intra-observer agreement, all measurements were performed twice at an interval greater than 2 weeks between evaluations.

**Fig. 1:**
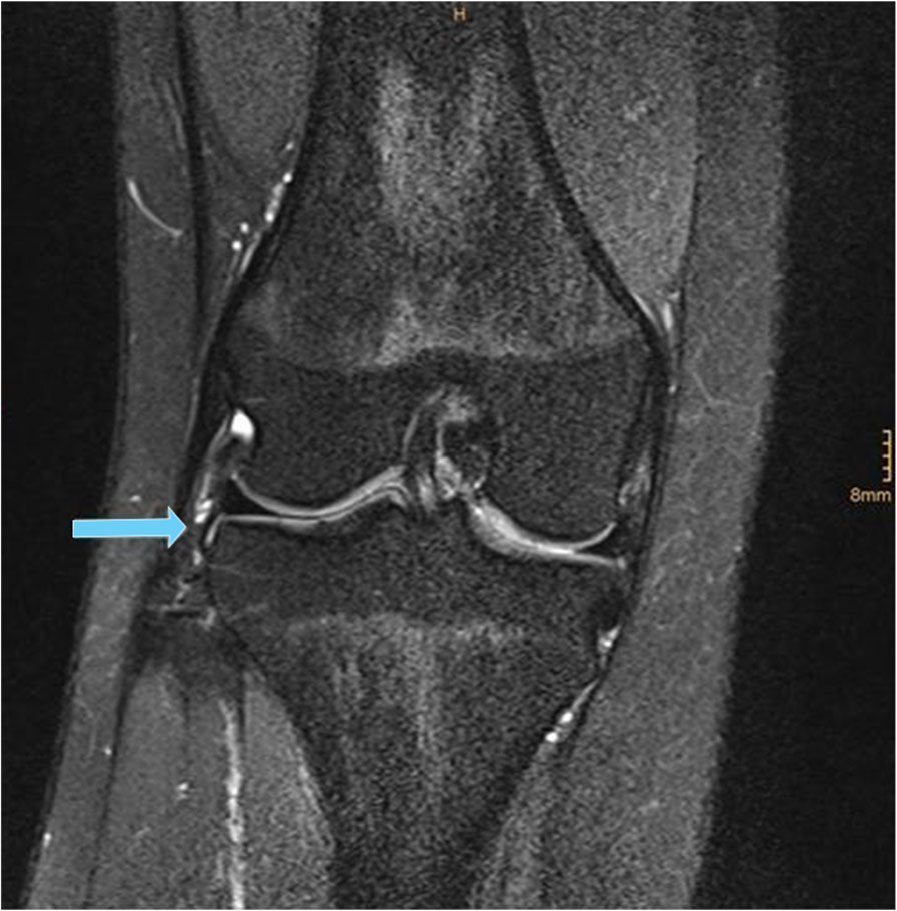
Tibial part (blue arrow) of the anterolateral ligament (ALL) as seen in 73.2% of the examined cases (coronal PD-weighted sequence with fat saturation)

The analysis to identify the ALL was performed in a manner similar to previous research [14, 37]. The ALL was defined as a low-signal band with its origin at the postero-proximal region of the femoral epicondyle, running in an antero-distal direction deep to the ITT (with optional fibers to the lateral meniscus; bifurcation point), crossing the lateral collateral ligament in its proximal third and inserting on the anterolateral tibia midway between Gerdy’s tubercle and the fibular head [8] (Fig. 1). Three parts of the ALL were defined and their presence assessed on a binary (yes/no) basis: a) femoral part, b) meniscal part, c) tibial part. The ALL was assessed only when present and clearly seen on both axial and coronal sequences under direct cross-referencing of images.

**Fig. 2:**
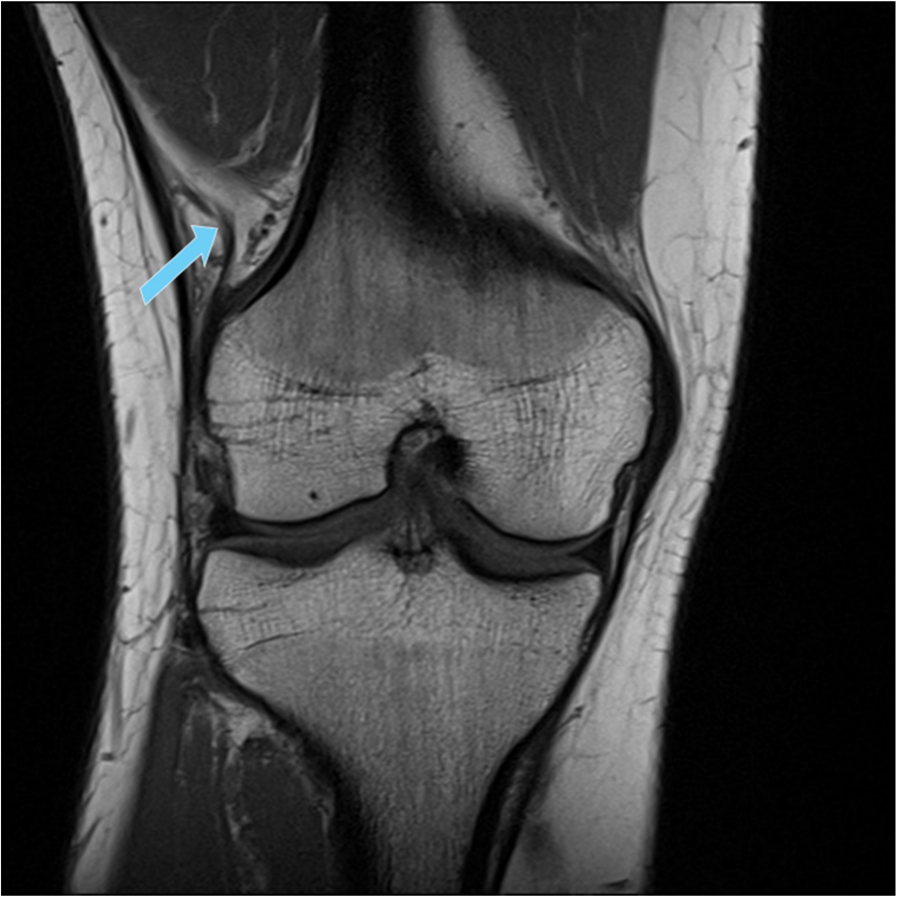
Deep attachments of the iliotibial tract (ITT) to the distal femur (blue arrow) as seen in 60.6% of the examined cases (coronal T1-weighted sequence)

The deep attachments of the ITT at the distal femur were also evaluated on a binary (yes/no) basis and further subcategorized according to the literature in three sub-structures: insertion near the septum, supracondylar insertion and retrograde insertion [8, 17, 23]. Furthermore, the presence of the capsulo-osseus layer of the ITT, defined as deep fibers running from the region of the Kaplan fiber complex to the anterolateral tibia, was examined [8, 17, 23, 28, 38] (Fig. 2).

After data analysis, a consensus meeting of the two radiologists was conducted to discuss discrepant findings.

### Statistics

Descriptive statistics for visibility of the above-mentioned structures are given for the consensus rating. Cohen’s kappa and 95% confidence intervals (95% CI) were determined as a measure of inter- and intra-observer reproducibility. In addition, we calculated the agreement separately for the categories “visible” and “not visible” as suggested by Cicchetti and Feinstein [2]. A value of 0.70 for Cohen’s kappa was deemed the threshold for substantial reproducibility [22]. Sample size considerations are based on a power analysis for a Pearson correlation as an approximation of Cohen’s kappa coefficient. Power analysis for Cohen’s kappa was not available in common power analysis software packages. An observed correlation coefficient of 0.824 in a sample of 70 cases was sufficient to demonstrate that the 0.70 threshold was exceeded with alpha = 0.05 and beta = 0.20 (one-sided). Power analysis was performed with G*Power 3.1.9.2.

## Results

Seventy-one patients (29 women, 40.8%) with an average age of 44.7 ± 14.1 years met the inclusion criteria.

In 62.0% of the cases the femoral part of the ALL was visible, whereas visibility of the meniscal and the tibial part of the ALL was 0% and 73.2%, respectively. The deep attachments of the ITT to the distal femur were detected in 60.6% of the cases. However, in none of the patients was it possible to further distinguish these fibers as ‘insertion near septum’, ‘supracondylar insertion’ or ‘retrograde insertion’. Visibility of the capsulo-osseous layer of the ITT was 0%.

Inter-observer agreement was high. Cohen’s kappa was 0.97 (95%CI: 0.9–1.00) for the tibial part of the ALL and 0.76 (95%CI: 0.61–0.92) for the femoral part of the ALL. As previously mentioned, neither of the two observers was able to detect the meniscal part of the ALL in any of the patients (100% absolute agreement). Therefore, it was not possible to calculate Cohen’s kappa for this variable. For the deep attachments of the ITT to the distal femur Cohen’s kappa was 0.94 (95%CI: 0.86–1.00). As the capsulo-osseous layer of the ITT was not visible to either of the observers in 100% of the cases, it was also not possible to calculate Cohen’s kappa for that parameter. For each of the investigated parameters absolute agreement between the observers was at least 88%. For further details, see Table 1.

**Table 1:** Inter- and intra-observer reproducibility results for the different parts of the ALL and the deep ITT structures

	Visibility	Inter-observer		Intra-observer	
	%	Cohen’s kappa (95% CI)	Absolute Agreement [%]^a^: Total / not visible/visible	Cohen’s kappa (95% CI)	Absolute Agreement [%]^a^: Total / not visible / visible
Femoral part ALL	62.0	0.76 (0.61–0.92)	88.7 / 85.2 / 90.9	0.62 (0.43–0.81)	83.1 / 75.0 / 87.2
Meniscal part ALL	0.0	n.c.	100 / 100 / 100	n.c.	100 / 100 / 100
Tibial part ALL	73.2	0.97 (0.9–1.00)	98.6 / 97.4 / 99.0	0.85 (0.70–0.91) 0.846 (0.701–0.911)	94.4 / 88.2 / 96.3
Deep attachments of the ITT to the distal femur	60.6	0.94 (0.86–1.00)	97.2 / 96.4 / 97.7	0.94 (0.86–1.00)	97.2 / 96.3 / 97.7
Capsulo-osseous layer of the ITT	0.0	n.c.	100 / 100 / 100	n.c.	100 / 100 / 100

Table 1 Inter- and intra-observer reproducibility results for the different parts of the anterolateral ligament (ALL) (femoral, meniscal, tibial) and the deep iliotibial tract (ITT) structures (deep attachments of the ITT to the distal femur, capsulo-osseous layer of the ITT). 95% CI: 95% confidence interval; n.c., not calculated

^a^The percentage (%) of absolute agreement is reported across all categories and separately for the categories “not visible” and “visible”

Regarding intra-observer agreement, Cohen’s kappa was 0.62 (95%CI: 0.43–0.81) for the femoral part of the ALL, 0.85 (95%CI: 0.70–0.91) for the tibial part of the ALL and not quantifiable for the meniscal ALL part. For the deep attachments of the ITT to the distal femur Cohen’s kappa was 0.94 (95%CI: 0.86–1.00). As the capsulo-osseous layer of the ITT was not visible in 100% of the cases, it was also not possible to calculate Cohen’s kappa for that parameter. For each of the investigated parameters absolute agreement between the two time points was at least 83% (Table 1).

## Discussion

The primary finding of the present study is good MRI visibility of the ALL and the deep fibers of the ITT with substantial and inter- and intra-observer agreement. The femoral and tibial parts of the ALL were visible in 62% and 73.2% of the cases, respectively, whereas the deep attachment of the ITT to the distal femur was detected in 60.6% of the cases. The capsulo-osseous layer of the ITT could not be assessed in any of the cases.

Several previous studies have dealt with MRI visibility of the ALL [9, 12–14, 20, 21, 24, 30, 37]. However only limited knowledge exists about inter-observer reproducibility [7, 13, 14, 37]. Inter- and intra-observer reliabilities of the ALL have been reported to range between 0.69–1.00 and 06–1.00, respectively [7, 13]. These results are in line with the present inter- and intra-observer agreements of 88% and 83%. In addition to the visibility of the different structures, an attempt was made to compare the descriptive values to the different parts of the ALL and the findings made in the above-mentioned studies. However, only three studies differentiated between the different parts of the ALL [12, 14, 24]. Visibility of the femoral (62%) and tibial part (73.2%) of the ALL was well consistent with previous studies. However, compared to the above-mentioned studies, the meniscal part of the ALL was detected less frequently. The reason for this discrepancy remains unclear. Other previous studies dealt with the distinct subject of ALL co-injury rates in patients with ACL rupture. However, this is beyond the scope of the present investigation [3, 10, 15, 27].

To the best of our knowledge, there exist no previous studies that deal with MRI visibility of the deep structures of the ITT. This is somewhat surprising because these structures were described from an anatomic and a biomechanical point of view many years ago and were considered important for anterolateral knee stability [17, 23, 28, 38]. In the present study, substantial agreement was found for MRI visibility of the deep structures of the ITT. The deep attachment of the ITT to the distal femur was detected in 60.6% of the cases. It was not possible to further distinguish these fibers as ‘insertion near septum’, ‘supracondylar insertion’ or ‘retrograde insertion’, as defined by Lobenhoffer [23]. It is noteworthy that in the present investigation the capsulo-osseous layer of the ITT could not be assessed in any of the cases. A possible explanation for this undifferentiability might be the immediate close proximity of many other structures like the superficial ITT or the tibial part of the ALL at the proximal anterolateral tibia. It seems reasonable that the limitations in the spatial resolution of MRI as compared with anatomic dissection [30] inhibit sufficient discrimination between a) the distal ALL, b) the superficial ITT and c) the capsulo-osseous layer of the ITT.

This work has some limitations. This was a retrospective investigation with the weaknesses typically associated with such studies. Although strict inclusion and exclusion criteria were applied, it would have been even better to include subjects 100% free of knee complaints. The subjects included in the present retrospective analysis were free of major knee lesions, but must have had some knee complaints as a prerequisite for MRI examination.

The major strength of the present study is that MRI visibility was investigated for the first time for both the ALL and the deep structures of the ITT in one single study, including a large cohort of patients and using a 3 Tesla MRI. Moreover, inter- and intra-observer reproducibility, which have not been reported in previous studies, were assessed. Compared to other studies, only healthy participants with no ligamentous lesions where included and examined.

Since the assessment and subsequently the treatment of an injured structure is preferably done after correct radiological representation, the clinical value of the present data is high. This is especially true, since injuries of the anterolateral structures are often seen with concomitant ACL ruptures [6]. Whether and how the anterolateral structures should be addressed at the time of ACL reconstruction is the subject of ongoing discussion [1, 8, 16, 25, 34]. Taken together the results of the present study substantially expand the current scientific insights into MRI visibility of the anterolateral structures of the knee and increase knowledge and safety in the field of anterolateral knee reconstruction.

## Conclusion

It can be concluded that the presence of the anterolateral structures of the knee can be determined using MRI with substantial inter- and intra-observer agreement. This is true for both the ALL and the deep structures of the ITT, but not for the capsule-osseous layer.

## Data Availability

The datasets used and/or analysed during the current study are available from the corresponding author on reasonable request.

## Abbreviations

ITT: Iliotibial Tract
ALL: Anterolateral Ligament
MRI: Magnetic Resonance Imaging
